# The potential mechanism of action of gut flora and bile acids through the TGR5/TRPV1 signaling pathway in diabetic peripheral neuropathic pain

**DOI:** 10.3389/fendo.2024.1419160

**Published:** 2024-11-15

**Authors:** Peng Chen, Xian Jiang, Jia Fu, Cehua Ou, Yao Li, Jing Jia, Changli Liao

**Affiliations:** ^1^ Department of Pediatrics, Southwest Medical University, Luzhou, Sichuan, China; ^2^ Department of Anesthesiology, Luzhou People’s Hospital, Luzhou, Sichuan, China; ^3^ Department of Pain Management, The Affiliated Hospital, Southwest Medical University, Luzhou, Sichuan, China; ^4^ Department of Science and Technology, Southwest Medical University, Luzhou, Sichuan, China; ^5^ Department of Anesthesiology, The Affiliated Hospital, Southwest Medical University, Luzhou, Sichuan, China; ^6^ Anesthesiology and Critical Care Medicine Key Laboratory of Luzhou, The Affiliated Hospital, Southwest Medical University, Luzhou, Sichuan, China

**Keywords:** diabetic peripheral neuropathic pain, gut flora, bile acids, TGR5, TRPV1

## Abstract

Diabetic peripheral neuropathic pain (DPNP) is a major complication of diabetes that markedly affects the quality of life and health status of patients. Recent studies have investigated the potential regulatory influence of gut flora and bile acids on DPNP via the TGR5/TRPV1 signaling pathway. Dysbiosis of the gut flora not only directly affects bile acid metabolism but also significantly correlates with diabetes-associated neuropathy through interactions with the bile acid receptor TGR5 and the ion channel TRPV1. This review describes how alterations in the gut flora and bile acid metabolism contribute to the pathogenesis of DPNP through the TGR5/TRPV1 signaling pathway, revealing potential applications for this pathway in DPNP management. Furthermore, experimental and clinical studies have demonstrated the modulation of gut flora and bile acid metabolism as well as targeting the TGR5/TRPV1 signaling pathway as an innovative therapeutic approach. Further studies are warranted to elucidate the underlying mechanism and develop treatment modalities based on gut flora regulation and signaling pathway interventions, thus providing novel insights and approaches for DPNP therapy.

## 1 Introduction

Diabetic peripheral neuropathic pain (DPNP), which is a common and challenging complication of diabetes mellitus, is mainly caused by diabetes-induced nerve damage and manifests as symmetrical peripheral neuropathic pain in the distal limbs, including mononeuralgia, brachial neuralgia, and lumbosacral neuralgia (1). Current studies have indicated that the prevalence of DPNP among patients with diabetes is as high as 22.8%–31.7% (2, 3). DPNP not only reduces the quality of life of patients but also increases the risk of cardiovascular events and overall mortality (2, 3). The prevalence of DPNP and its impact on patient quality of life are especially evident in patients with type 2 diabetes mellitus (T2DM) (4), with theories in a review supporting the influence of gut flora on DPNP (5). The review outlined the correlation between T2DM and intestinal flora, elucidating its underlying pathological mechanism. Building on this review, the mechanism of action between intestinal flora and bile acid in DPNP through the Takeda G-Protein Receptor 5 (TGR5)/ Transient Receptor Potential Vanilloid Subtype 1 (TRPV1) signal pathway is discussed in detail, to provide theoretical basis for the prevention and treatment of DPNP from the perspective of intestinal flora and bile acid. Diabetic peripheral neuropathy encompasses sensory, motor, and autonomic neuropathy. Implicated causes of peripheral nerve damage include oxidative stress damage; accumulation of sorbitol; advanced glycosylation end products; and a disturbance of hexosamine, protein kinase C, and polymerase pathways. Neurovascular impairment with poor repair processes and endothelial dysfunction have also been implicated (6). However, there are some review papers and retrospective studies that showed the connection between bilirubin serum levels and T2DPNP. The human gut flora is a highly diverse ecosystem comprised of a myriad of bacteria; further, there are theories that metabolites interact with the nervous system in a bidirectional manner through the gut–brain axis (7). Accordingly, gut flora could play different regulatory roles in neurological disorders.

Bile acids, which are synthesized by the liver, are crucial organic molecules that not only play a central role in lipid digestion and absorption but also engage in a complex bidirectional interaction with intestinal microbial communities (8).This interaction affects the diversity and balance of intestinal flora by regulating bile acid metabolism and synthesis (8). Notably, bile acids are involved in numerous physiological processes, including the pathogenesis of diabetic neuropathy, through TGR5 and other receptors (9, 10). In addition, our research group found that the diversity and abundance of intestinal flora in DPNP rats were affected; especially, the relative abundance of Clostridium, Dorea, and Streptococcus increased significantly in the DPNP group. Through a preliminary assessment of serum metabolism in DPNP rats, we found a significant enrichment in the secondary bile acid, deoxycholic acid, within the secondary bile acid biosynthesis pathway. This was significantly upregulated in the serum of the DPNP model group, which suggests a possible relationship between intestinal flora, bile acid, and DPNP. This review article summarizes the potential mechanism underlying DPNP regulation by intestinal flora through bile acid and TGR5 receptors as well as explores the application prospects of this signaling pathway in DPNP therapy. Specifically, this review summarizes the latest research in order to help elucidate the mechanisms underlying the interplay among intestinal flora, bile acids, and DPNP. Furthermore, it explores potential treatment approaches based on this mechanism, which could provide valuable insights for future clinical practice and research endeavors.

## 2 Intestinal flora, bile acids, and diabetes

### 2.1 Mechanism of action of intestinal flora

There has been increasing interest in the role of the gut microbiota in the pathogenesis of diabetes. Studies have demonstrated distinct disparities in the gut microbiota composition between individuals with and without diabetes. For example, patients with diabetes have reduced numbers of beneficial gut bacteria, including *Lactobacillus*, *Bifidobacterium*, and *Fecalibacterium prausnitzii*, and an increased number of certain flora potentially associated with diseases, including *Escherichia coli*, *Enterococcus*, and *Clostridium* (11, 12). This shift in microbial composition not only mirrors the diabetes-related alterations in intestinal milieu but may also contribute toward disease onset.

**Figure 1:**
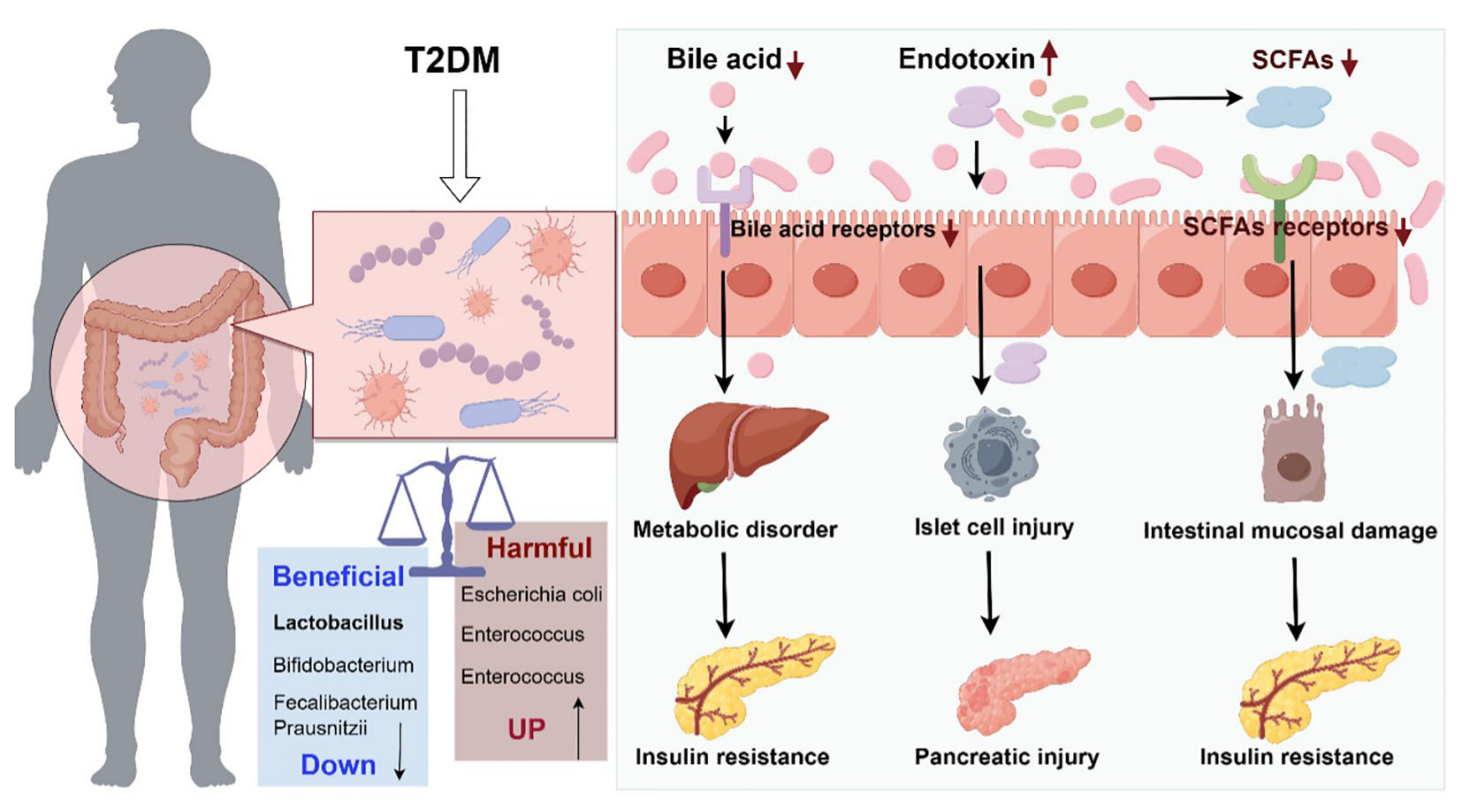
Schematic diagram of the role of intestinal flora dysregulation in diabetes (Drawn using Figdraw, color in RGB). The decrease of probiotics and increase of harmful bacteria in the gut tract leads to microecological dysregulation of gut flora. Dysbiosis of gut flora causes abnormal bile acid metabolism and abnormal activation of bile acid receptors, leading to metabolic disorders and insulin resistance. Dysbiosis of gut flora leads to reduced gut barrier function and increased endotoxin penetration, causing islet cell and pancreatic damage. Dysbiosis of gut flora leads to abnormal short-chain fatty acid metabolism, which acts on short-chain fatty acid receptors, causing small gut mucosal damage and insulin resistance.

Gut flora has been found to be strongly associated with T2DM (4). The following hypotheses have been proposed (**Figure 1**):

#### 2.1.1 Bile acid theory

As an important intermediary substance in intestinal and hepatic metabolism, the metabolic process of bile acids is significantly affected by intestinal flora. Disturbances in intestinal flora may lead to reduced production of secondary bile acids, which in turn reduces the activation of bile acid receptors (e.g., TGR5 and FXR) (13, 14), and thus affects glucose metabolism and possibly contributes toward the development of T2DM (15, 16). The activation of bile acid receptors is crucially involved in regulating glucose and lipid metabolism as well as improving insulin sensitivity.

#### 2.1.2 Short-chain fatty acid theory

Intestinal flora can also influence the metabolic state of the host through the production of short-chain fatty acids (e.g., butyric, propionic, and acetic acids). Short-chain fatty acids are an important source of energy; additionally, a reduced number of bacteria that produce short-chain fatty acids results in reduced levels of these fatty acids (17, 18), which impairs pancreatic islet cell function and reduces insulin sensitivity (19) through a decreased ability of the intestines to respond to anti-inflammatory reactions (20); moreover, it results in a weakened ability to activate short-chain fatty acid receptors (21). In patients with diabetes, an abnormal number of short-chain fatty acid-producing bacteria may weaken these protective mechanisms, and thus result in T2DM development.

#### 2.1.3 Endotoxin theory

Dysbiosis of gut flora may result in reduced intestinal barrier function (22) and increased penetration of endotoxins (e.g., lipopolysaccharides), which can enter the circulation and elicit an inflammatory response, leading to insulin resistance (23) and impaired insulin signaling (24), thus contributing to the development of T2DM.

In summary, gut flora significantly influence the host's metabolic state through multiple mechanisms. These include the modulation of bile acid metabolism, generation of short-chain fatty acids, and regulation of endotoxin levels, which collectively influence the development of diabetes.

### 2.2 Biological role of bile acids

Bile acids can be classified as primary or secondary bile acids based on their origin. Primary bile acids are synthesized directly from cholesterol in hepatocytes, while secondary bile acids are produced through the conversion of primary bile acids by intestinal flora. Specifically, primary bile acids are secreted by hepatocytes and excreted into the intestine via the biliary system, where they are converted into secondary bile acids through the action of intestinal bacteria. Approximately 95% of secondary bile acids are reabsorbed at the end of the small intestine into the hepatic portal vein, where they are transported to the liver for use in a new secretion process, which is a process termed as the enterohepatic cycle of bile acids (25). Bile acids not only help regulate cholesterol homeostasis and promote the digestion and absorption of lipids but also act as signaling molecules involved in metabolic regulation (26). Specifically, bile acids are involved in glucose and lipid metabolism (27), and abnormal glucose metabolism may affect bile acid metabolism in patients with T2DM (28). *In vivo* studies have demonstrated a bidirectional relationship between bile acids and glucose metabolism as well as the involvement of bile acids in T2DM development. Another study found that bile acids can activate the bile acid receptor TGR5, and thus, improve glucose tolerance, insulin sensitivity, and energy metabolism (29). Taken together, these findings demonstrate the crucial role of bile acids in metabolic regulation.

## 3 Role of the TGR5/TRPV1 signaling pathway in DPNP

### 3.1 Overview of the TGR5/TRPV1 signaling pathway

TGR5 is a G protein-coupled receptor that binds bile acids and promotes cyclic adenosine 3',5'-monophosphate (cAMP) synthesis via adenylate cyclase, which subsequently activates the protein kinase A pathway and induces the expression of target genes (30). This process induces the production of type II iodothyronine deiodinase, which increases thyroxine synthesis and secretion, thus affecting glucose metabolism. Therefore, TGR5-expressing genes are considered to be closely linked to diabetes mellitus (31). TGR5 is expressed in the nervous system, including in neurons of the dorsal root nerve and in neuroglia (32), which are related to pain-related afferent nerve sensitization. TGR5 activation in neurons of the dorsal root nerve leads to neuronal hyperexcitability and dorsal horn neurotransmitter release (32). Additionally, bile acids can stimulate TGR5 to induce nociceptor sensitization in the intestinal nervous system, which induces mechanical hypersensitivity (33). For example, TGR5 receptors can play a role in mediating hypersensitivity to bladder distension (30), which further mediates the production of itching sensations. Pruritus can be induced by both histaminergic and non-histaminergic mechanisms, with bile acids being crucial factors in the latter (34). DPNP is characterized by pain resulting from an abnormal somatosensory system, which indicates a potential relationship among bile acids, the TGR5 receptor, and DPNP.

TRPV1 is a cation channel that is highly expressed in dorsal root ganglion neurons; additionally, it is closely associated with DPNP development (35). TRPV1 activation can contribute to the development of DPNP (36); contrastingly, inhibition of TRPV1 channels in the dorsal root ganglion can attenuate DPNP symptoms (37). TRG5 is closely associated with TRPV1 and may act upstream and downstream of the same signaling pathway and jointly participate in the onset and development of DPNP and other related conditions. TRG5 mRNA is exclusively co-expressed with TRPV1 in dorsal root ganglion innervating the bladder (30); additionally, TGR5 activates the cAMP-cAMP response element-binding protein (CREB) signaling pathway. Under cAMP stimulation, CREB activates TRPV1 promoter transcriptional activity, which in turn induces abnormal peripheral pain through activation of the downstream signaling protein kinase C (38). Activated TGR5 modulates itch and provide analgesia by regulating the expression of cation channels such as TRPV1 (39). Further exploration of this pathway would facilitate the elucidation of the mechanisms underlying DPNP and similar conditions as well as the identification of novel treatment targets.

### 3.2 Relationship between diabetes and TGR5/TRPV1 signaling pathway

TGR5 is expressed in various tissues, and its main function is to maintain blood sugar level and increase energy consumption. Activated TGR5 can up-regulate the production and secretion of glucagon-like peptide -1(GLP-1) in intestinal endocrine cells and improve glucose homeostasis (40). Activation of TGR5 can promote lipolysis and energy consumption (41) and promote metabolic improvement and advanced weight management (42). TGR5 shows a potential anti-diabetic effect, and TGR5 agonists become potential candidates for the treatment of type 2 diabetes, obesity and other metabolic diseases (43).

Type 1 diabetes is an autoimmune disease and TRPV1 is potentially associated with autoimmune abnormalities (44). Impaired muscle Ca^2+^ homeostasis in type 1 diabetic rats was found to be due to TRPV1-mediated attenuation of heat stress tolerance, and capsaicin or other therapeutic strategies that increase Ca^2+^ accumulation via TRPV1 may be more effective than heat therapy in type 1 diabetic patients (45). Continuous oral cilostazol treatment was effective in reducing the level of painful peripheral neuropathy in streptozotocin-induced type I diabetic rats, which may be related to denervation of sensory nerves in the epidermis of the hind paw of DM rats, with a significant reduction in TRPV-1-labeled penetrating nerve fibers (46). Lipid peroxidation products were found to trigger mitochondrial calcium inward flow and mitochondrial dysfunction in endothelial cells in diabetic patients and TRPV1 agonists, and TRPV1 knockout mice were protected from type 1 diabetes-induced endothelial dysfunction and impaired vascular regeneration after arterial injury (47), and TRPV1 activation may be involved in mediating the process of aberrant lipid metabolism that contributes to the onset of diabetes.TRPV1 channel activation plays a protective role in cardiac oxidative/nitrative stress, mitochondrial function, endothelial function, inflammation, and cardiac energy metabolism in diabetic models, and activation of TRPV1 channels can delay the progression of diabetic complications to a certain extent (48). In addition, activation of TRPV1 by capsaicin can mediate insulin signaling-independent glucose oxidation and ATP production in mouse skeletal muscle cells (49), and TRPV1 likewise has potential antidiabetic effects.

### 3.3 TGR5/TRPV1 cross-pathway affecting DPNP

Other signaling pathways exist that are cross-relational with TGR5/TRPV1 and influence the development of DPNP. It has been found that GPR177 in A-fiber sensory neurons drives diabetic neuropathic pain through WNT-mediated activation of TRPV1, and that GPR177 mediates the secretion of WNT5a from A-fiber DRG neurons into the cerebrospinal fluid (CSF), which is required for the maintenance of DNP (36). Increased P2X3 and TRPV1 activity may mediate the pre-inflammatory spinal cord cytokine release, causing DPNP to occur (50). Additionally α-lipoic acid (ALA) may modulate TRPV1 expression by affecting NF-κB, thereby reducing diabetic neuropathic pain (51). Inosine can attenuate diabetic peripheral neuropathy by modulating GLO1/AGEs/RAGE/NF-κB/Nrf2 and TGF-β/PKC/TRPV1 signaling pathways (52). And ropivacaine can affect peripheral neuropathy in streptozotocin-diabetic rats via TRPV1-CGRP pathway (53). It can be seen that there exists a pathway that crosses with TGR5/TRPV1, which can also affect DPNP development by activating TRPV1.

### 3.4 Gut flora, bile acids, and the TGR5/TRPV1 signaling pathway

**Figure 2:**
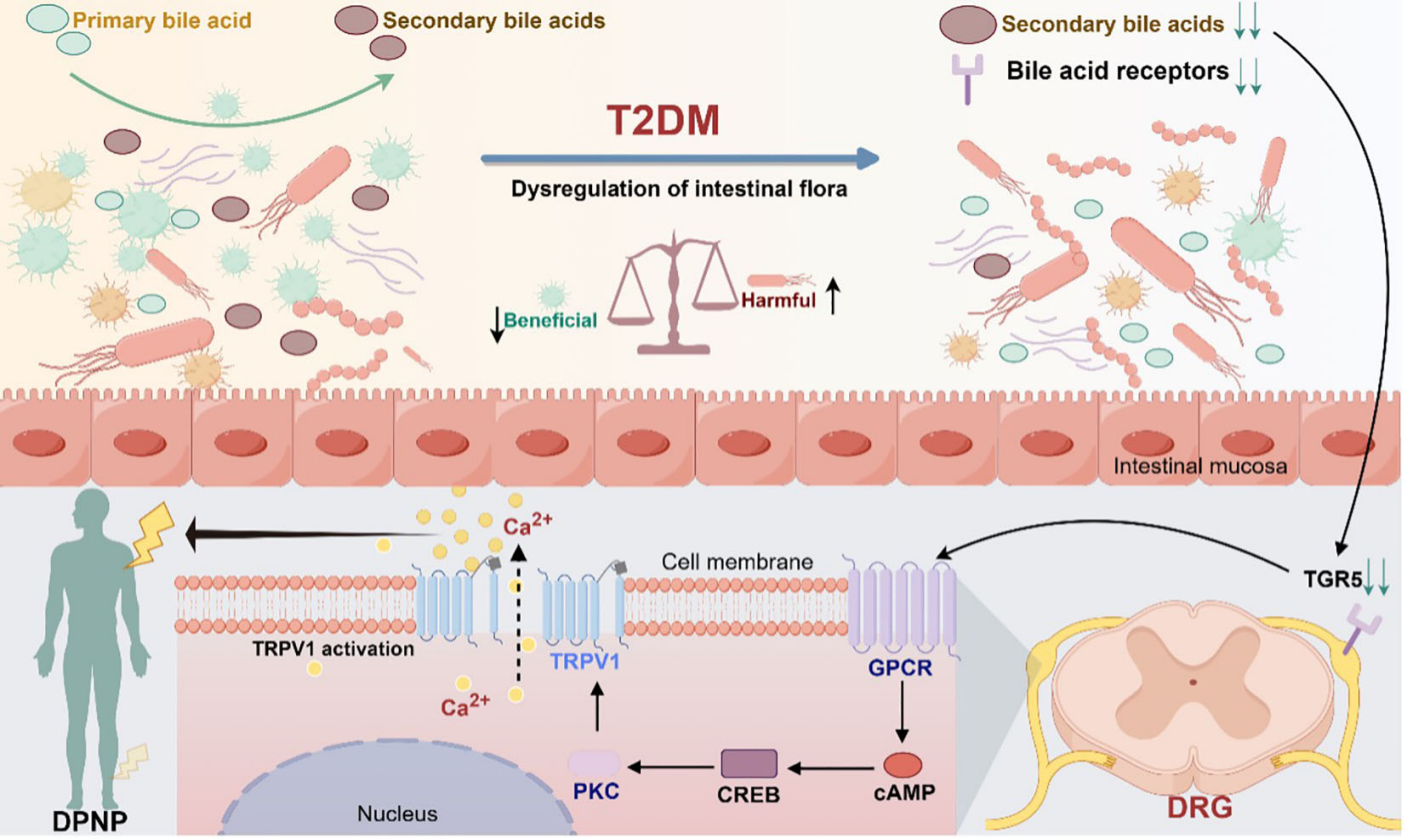
Modulation of TGR5 receptors and TRPV1 ion channels in gut microbiota mediating diabetic peripheral neuropathic pain (Drawn using Figdraw, color in RGB). Dysbiosis of gut flora causes abnormal bile acid metabolism and activates the bile acid receptor TGR5, which promotes the synthesis of cAMP through adenylate cyclase. Under the stimulation of cAMP, the cyclic adenosine monophosphate response element-binding protein CREB activates the transcriptional activity of the TRPV1 promoter, which in turn activates the downstream signaling protein kinase C. Through the downstream pathway, it can induce abnormal pain in the peripheral area.

Taken together, patients with diabetes undergo changes in the gut flora composition, which are influenced by environmental factors or their own metabolism. As a result, there are alterations in the intestinal bacterial metabolism of primary bile acids, leading to abnormalities in the metabolism of secondary bile acids, which may, in turn, abnormally activate the cAMP-CREB signaling pathway through action on the TGR5 receptor. This abnormal activation could cause abnormal expression and transcription of TRPV1, leading to downstream signaling that triggers peripheral neuropathic pain. This mechanism describes the entire process from microbial changes to neuropathy in patients with diabetes and provides novel insights into the pathophysiology of DPNP (**Figure 2**).

## 4 Experimental studies and clinical observations

Experimental and clinical studies have provided strong evidence regarding the interaction among gut flora, bile acids, and the TGR5/TRPV1 signaling pathway, as well as their role in the pathogenesis of DPNP. Studies on gut flora have reported that an increase in specific microorganisms such as *Desulfovibrio vulnificus* can increase secondary bile acid production and cecal hydrophobicity, which affects bile acid metabolism and function (54).

*In vitro* experimental studies have shown that application of TRG5 agonists to bladder-innervating dorsal horn neurons increase intracellular calcium levels, with this process being mediated by TRPV1 channels (55). This suggests a strong link between TGR5 and TRPV1 with respect to neuronal activity and nociceptive transmission. Furthermore, serum lysophosphatidic acid induces limbic activation of heterologously expressed TRPV1, which subsequently regulates neuronal reactivity and sensations such as itching and pain (56). Moreover, mouse studies have demonstrated that TGR5 activation induces itch responses via TRPV1 (32). Taken together, these findings demonstrate that TRG5 and TRPV1 are closely related to neurons; additionally, the TRG5/TRPV1 signaling pathway is crucial for regulating neurosensory sensations, which may be involved in DPNP development.

TGR5 is highly expressed in cells such as monocytes and macrophages, and TGR5 activation produces potent systemic anti-inflammatory properties (57). Dermatitis-lesioned mice have shown TGR5 protein activation and consistently increased TRPV1 expression (58). Furthermore, there is generally reduced expression of inflammatory markers in mice following bilirubin feeding (58). Bile acid synthesis in the liver is correlated with the severity of itching, while bile pigments reduce bile acid levels, decrease the expression of inflammatory markers, and relieve itching (59). Therefore, bile acids may regulate the TRG5/TRPV1 signaling pathway.

Taken together, there is evidence indicating the potential role of the gut flora-bile acid-TGR5/TRPV1 signaling pathway in regulating DPNP development. This study offers new research avenues and identifies potential therapeutic targets for diabetes and its complications. Moreover, these findings underscore the intricate interplay between gut flora, metabolites such as bile acids, and nervous system. They offer a crucial scientific foundation for elucidation of disease mechanisms and development of novel therapeutic approaches.

## 5 Prevention and treatment of DPNP

The current treatment strategy for the prevention and treatment of DPNP mainly relies on traditional analgesic drugs such as glucose control, amitriptyline, duloxetine, pregabalin, and gabapentin. Some researchers (60) have proposed the idea of combining multiple medications for DPNP treatment; however, not all these medications can completely treat or improve DPNP symptoms. Therefore, it is important to develop novel therapeutic approaches and targets. We found that intestinal flora, bile acids, bile acid receptor signaling pathways (TGR5 and TRPV1), and DPNP are closely related, and that abnormalities in the composition of intestinal flora in diabetic patients cause abnormalities in bile acid metabolism, which in turn lead to abnormal activation of the relevant bile acid receptor signaling pathways, thus inducing DPNP. Based on this, we believe that regulating the intestinal flora through interventions such as probiotic or prebiotic supplementation, dietary fiber intake, and even fecal transplantation, could correct the intestinal flora disorders in patients with DPNP, which may ameliorate the occurrence and development of DPNP from the source. Currently for regulating the acute effects of this pathway on DPNP is not clear, the role of this pathway on DPNP is more reflected in the chronic effects.

### 5.1 Regulation of gut flora and bile acids

Dysbiosis of gut flora in patients with diabetes leads to a reduction in probiotics and prebiotics. Accordingly, supplementation with beverages containing probiotics and/or prebiotics can improve T2DM symptoms (61) by restoring the intestinal microenvironment and ameliorating glucose tolerance abnormalities (62). The use of antibiotics to alter the composition of gut flora has been found to alter blood glucose levels and glucose tolerance in animal models (63). Moreover, *Lactobacillus reuteri J1* can alter the composition of gut flora and bile acids, which can be involved in obesity treatment (64). Additionally, dietary fiber supplementation can increase the content of beneficial bacteria in the gut, which in turn establishes functionally active intestinal flora and regulates energy metabolism to a certain extent in order to improve T2DM symptoms (5). In mice, oligofructose supplementation for modulating gut flora has been found to enrich bacteria involved in 6α-hydroxylated bile acid production, which can activate the TGR5-GLP1R axis to improve body weight and metabolism in mice (65). Fecal flora transplantation, i.e., transplantation of gut probiotics from healthy populations to patients, has also demonstrated potential in reshaping the intestinal microecological balance and improving insulin sensitivity (66).Additionally, direct supplementation with bile acids such as ursodeoxycholic acid (67) and glycine ursodeoxycholic acid (68) may facilitate regulation of bile acid metabolism and protection of pancreatic islet β-cells (69); however, its antidiabetic effects remain unclear (70). The regulation of intestinal flora and bile acid metabolism may inform prevention and treatment strategies for T2DM and DPNP (68).

### 5.2 Regulation of TGR5 receptors and TRPV1 ion channels

Pharmacological modulation of receptors is a valuable approach for developing treatments for various diseases (71). This is because the specificity of the central pathway of sensory input is driven by a combination of receptors and ion channels expressed on sensory endings, which allow differentiation between different stimuli. Specific targeted modulation of the TGR5 receptor on sensory nerve endings is a potential treatment strategy for DPNP. TGR5 agonists have been identified as potential targets for the treatment of T2DM and other metabolic disorders, including obesity, by modulating GLP-1 secretion and increasing energy expenditure in the adipose tissue (43). The downstream TRPV1 channel of TGR5 is involved in the regulation of several important physiological and pathological processes; accordingly, TRPV1 is considered a promising therapeutic target for various diseases, including diabetes mellitus (72). Rat studies have indicated that α-lipoic acid can attenuate neuropathic pain in diabetic rats by downregulating TRPV1 receptors via NF-κB (51) and inhibiting TRPV1 channels (73). Additionally, the antidepressant mirtazapine has shown beneficial effects in diabetes-induced nociceptive hypersensitivity effect, which are achieved by enhancing the inhibitory effect on TRPV1 (74). Taken together, TGR5 and TRPV1 are potential key targets for the treatment of DPNP.

### 5.3 Combination therapy of gut flora, bile acids and TGR5/TRPV1

Monotherapy directly targeting TGR5/TRPV1 only improves DPNP symptoms and fails to correct the etiology of the disease Not all medications can completely improve symptoms in patients with DPNP (60). Compared with monotherapy, combination therapy involving intestinal flora regulation and bile acids can intervene in the development of diabetes mellitus and DPNP from the source, and the regulation of intestinal flora and bile acids may correct metabolic disorders and improve insulin resistance, which has a better therapeutic effect.

## 6 Conclusion and outlook

This review discusses the role of the gut flora-bile acid-TGR5/TRPV1 signaling pathway in the development of DPNP. An imbalance in intestinal flora not only affects bile acid metabolism, which, in turn, affects the onset and development of diabetes mellitus, but also is directly related to the development of diabetes-related neuropathy. Novel therapeutic strategies are in development for DPNP by regulating intestinal flora and bile acids as well as targeting TGR5 and TRPV1. We recognize the shortcoming of literature review articles in relation to meta-analysis and systematic reviews. Future studies are warranted to further elucidate the specific mechanisms underlying the interactions among gut flora, bile acids, and the TGR5/TRPV1 signaling pathway, as well as their involvement in DPNP. In addition, it is important to develop novel therapeutic approaches and drugs based on this mechanism, specifically through fecal flora transplantation, probiotic and prebiotic supplementation, and agonists/antagonists targeting TGR5 and TRPV1, for patients with DPNP. However, whether compensatory mechanisms exist in the gut flora or bile acid signaling pathway to attenuate or enhance the effects on the TGR5/TRPV1 pathway is unclear, and more research is needed for the practical application of this pathway. Accordingly, there is a need for clinical trials to validate the efficacy and safety of these treatment strategies. The ultimate treatment goal is to improve both the symptoms and quality of life of patients with DPNP through modulation of diabetes-related metabolic disorders through these combined treatment strategies.
